# EGCG alleviates PM2.5-induced lung injury via activation of PPAR-γ to suppress inflammation and oxidative stress

**DOI:** 10.3389/fphar.2025.1695785

**Published:** 2025-11-13

**Authors:** Kai Liu, Dean Wu, Chunyan Li, Dongxia Tang, Chuanwei Xu, Tongjing Li

**Affiliations:** 1 Department of Oncology, The First Affiliated Hospital of Henan Polytechnic University (Jiaozuo Second People’s Hospital), Jiaozuo, China; 2 Department of Respiratory and Critical Care, The Third People’s Hospital of Gansu Province, Lanzhou, China; 3 Department of Clinical Nutrition, The First Affiliated Hospital of Henan Polytechnic University (Jiaozuo Second People’s Hospital), Jiaozuo, China

**Keywords:** PM2.5, lung injury, EGCG, PPAR-gamma, oxidative stress, natural compounds

## Abstract

Fine particulate matter (PM2.5), a prevalent air pollutant, induces pulmonary injury by triggering inflammatory responses and oxidative stress, leading to cellular damage and tissue disruption. Epigallocatechin gallate (EGCG), a natural polyphenol compound derived from plants and known for its anti-inflammatory and antioxidant properties, has not been thoroughly investigated regarding its protective role and underlying mechanisms against PM2.5 triggered lung injury. This study employed a murine model of lung injury triggered by PM2.5 and the BEAS-2B cells to evaluate the effects of EGCG. We measured the levels of inflammatory cytokines and oxidative stress markers, alongside examining the expression of peroxisome proliferator-activated receptor gamma (PPAR-γ) and its downstream effectors nuclear factor-kappa B (NF-κB) and heme oxygenase-1 (HO-1). PM2.5 exposure induced pathological alterations in mouse lung tissues, including inflammatory cell infiltration and alveolar wall thickening. Both *in vivo* and *in vitro*, PM2.5 elevated pro-inflammatory cytokines (IL-1β, IL-6, and TNF-α), increased reactive oxygen species and malondialdehyde levels, and reduced the activity of antioxidant enzymes (catalase and superoxide dismutase). Furthermore, PM2.5 suppressed PPAR-γ expression, activated NF-κB signaling, and decreased HO-1 expression. Pretreatment with EGCG effectively upregulated PPAR-γ expression, subsequently inhibited NF-κB activation, and enhanced HO-1 activity, thereby attenuating inflammatory and oxidative stress responses. Critically, co-administration of the PPAR-γ antagonist T0070907 partially reversed the EGCG’s protective actions, as evidenced by the renewed escalation in cytokine production and oxidative damage. Our findings demonstrate that EGCG, a promising plant-derived bioactive compound, may ameliorate PM2.5 related lung injury by modulating PPAR-γ, which consequently mitigates inflammatory signaling and oxidative imbalance. This study elucidates a novel pharmacological mechanism by which EGCG ameliorates air pollution-induced lung injury.

## 1 Introduction

Fine particulate matter (PM2.5) comprises airborne particles possessing an aerodynamic diameter below 2.5 μm (Wang and Liu, 2023), exerts direct harmful effects on the respiratory tract and epithelial cells, triggering diverse toxic responses that contribute to various respiratory diseases (Sang et al., 2022). Acute high-level PM2.5 exposure directly contributes to pulmonary inflammation and structural tissue damage (Yue et al., 2019). Consequently, mitigating PM2.5-induced respiratory injury represents a critical public health challenge.

Inflammation and oxidative damage are recognized as central mechanisms in lung injury triggered by PM2.5 (Gao et al., 2022). Upon inhalation, PM2.5 particles penetrate deep into the distal airways, depositing in terminal bronchioles and alveoli. There, they stimulate epithelial cells, prompting a swift secretion of pro-inflammatory mediators, including interleukin-1β (IL-1β), interleukin-6 (IL-6), and tumor necrosis factor-α (TNF-α) (Liu et al., 2022). This triggers an inflammatory cascade that disrupts subcellular integrity and culminates in pulmonary inflammation and tissue damage. These processes lead to overproduction of reactive oxygen species (ROS) and compromise the intracellular redox defenses. The synergistic interaction between oxidative stress and inflammatory cytokines further aggravates lung tissue damage and exacerbates a range of respiratory conditions (Hou et al., 2024). Studies have shown that particulate matter can induce pulmonary emphysema and airway inflammation in mice through mechanisms closely linked to inflammatory signaling and redox imbalance (Wang et al., 2022). Therefore, effectively controlling inflammatory and oxidative stress responses presents a promising therapeutic strategy for alleviating PM2.5-induced respiratory impairment and dysfunction.

Peroxisome proliferator-activated receptor-γ (PPAR-γ) plays crucial regulatory roles anti-inflammatory and immune responses (Kökény et al., 2021). Activation of PPAR-γ regulates key inflammatory signaling cascades, including the NF-κB signal, leading to inhibited pro-inflammatory cytokine secretion and reduced tissue inflammation (Carvalho et al., 2021). Moreover, PPAR-γ activation bolsters cellular antioxidant defenses by inducing heme oxygenase-1 (HO-1) expression, thereby elevating the activity of key enzymes like superoxide dismutase (SOD) and catalase (CAT). This coordinated enhancement facilitates the removal of surplus ROS and improves resistance to environmental stressors (Zhang et al., 2022). Consequently, Targeted pharmacological activation of PPAR-γ offers a promising strategy for treating inflammatory lung conditions by concurrently inhibiting NF-κB signaling and upregulating HO-1 expression, which collectively reduce inflammatory responses and restore redox homeostasis (Lee et al., 2024).

Natural compounds derived from plants have demonstrated considerable potential in lung injury, and their underlying mechanisms of action continue to be actively investigated (Chen et al., 2025). For instance, A previous study demonstrated that curcumin, a plant-derived polyphenol, alleviated PM2.5-induced lung injury in mice by regulating the HO-1/CO/P38 MAPK signaling pathway, thereby mitigating oxidative stress and inflammatory responses (Huang et al., 2019). Similarly, quercetin, a natural flavonoid, was reported to protect against PM2.5-induced chronic lung injury and fibrosis in mice by activating the Nrf2-Keap1 signaling pathway to suppress ferroptosis, a novel form of regulated cell death linked to inflammation (Ding et al., 2024). Building on the broad potential of phytochemicals, Epigallocatechin gallate (EGCG), the principal bioactive catechin found in green tea, serves as the primary bioactive component of tea polyphenols and is the most prevalent form within the catechin subclass (Talib et al., 2024). EGCG exhibits potent antioxidant and anti-inflammatory activities, attributable to its distinctive molecular composition (Jin et al., 2025). Consistent with this, prior research has shown that EGCG alleviates lipopolysaccharide (LPS)-induced acute lung injury in murine models via attenuation of neutrophil and macrophage infiltration, decreased myeloperoxidase activity, and pro-inflammatory cytokine release inhibition (Wang et al., 2021). Moreover, in a rat model of silicosis, early intervention with EGCG was shown to attenuate pulmonary inflammation and exhibit a potential to prevent the progression of fibrosis, underscoring its broad therapeutic potential against particulate matter-induced lung injuries, albeit through mechanisms not yet fully elucidated (Adamcakova et al., 2023). Although EGCG’s broad protective effects are recognized, the specific role of the PPAR-γ signaling pathway in mediating its action against PM2.5-induced pulmonary damage remains to be investigated.

Accordingly, we employed a murine PM2.5 airway exposure model to mimic environmentally relevant lung injury and used BEAS-2B cells to explore EGCG’s protective mechanisms against pulmonary damage.

## 2 Materials and methods

### 2.1 Chemicals and reagents

EGCG (≥98% purity) was supplied by Beijing Solarbio Science & Technology Co., Ltd. (Beijing, China), while the PPAR-γ antagonist T0070907 was acquired from KKLmed Co., Ltd. (Shanghai, China). Detailed information regarding antibodies used for Western blot and experimental kits is available in Supplementary Tables S1, S2.

### 2.2 PM2.5 collection and preparation

PM2.5 was sampled adjacent to a high-traffic roadway in Lanzhou, China, followed by filter fragmentation and ultrasonication in double-distilled water. This elution process was repeated twice to maximize yield. The filtered solution was subsequently passed through an eight-layer sterile gauze to eliminate large particulate matter, lyophilized for 24–48 h (Shan et al., 2022; Shi et al., 2025). To confirm the nature and composition of the collected particulates, a representative portion of the filter sample was subjected to chemical analysis. The concentrations of key components, including water-soluble ions, metal elements, and organic carbon (OC)/elemental carbon (EC), were determined using ion chromatography, inductively coupled plasma mass spectrometry, and thermal-optical analysis, respectively. The detailed compositional data are listed in Supplementary Tables S3–S6.

### 2.3 Animal grouping and interventions

Eight-week-old male specific pathogen-free Balb/c mice were supplied by the Lanzhou Veterinary Research Institute, Chinese Academy of Agricultural Sciences. All animal procedures were approved by the Ethics Committee of the Second People’s Hospital of Jiaozuo (Approval No.: KY2025-07-080).

A total of 32 mice were assigned to four experimental groups randomly: (a) Control; (b) EGCG; (c) PM2.5-induced lung injury; (d) PM2.5 + EGCG. To induce lung injury, animals in groups (c) and (d) received daily intratracheal administration of a PM2.5 suspension (2 mg/mL, 50 μL; 100 μg total particulates) for 2 days (Ma et al., 2023; Shi et al., 2023). Control and EGCG-only mice were given an equivalent volume of sterile saline. Animals in the EGCG-treated groups (b and d) received EGCG via intraperitoneal injection (20 mg/kg) 3 times. The first dose was administered 24 h before the initial PM2.5 exposure, followed by additional doses 1 h before the two instillations. Saline was administered to control and PM2.5-only groups according to the identical dosing regimen (Shen et al., 2017; Almatroodi et al., 2020). All animals were euthanized 24 h following the last PM2.5 instillation under deep anesthesia induced by 0.5% sodium pentobarbital.

### 2.4 Collection and preservation of BALF and serum

Serum was collected via orbital enucleation, then centrifuged, aliquoted, and cryopreserved. Following thoracotomy, the left main bronchus was ligated followed by rapid excision of the left lung. Tissue segments designated for histopathological examination were fixed in 4% paraformaldehyde. Bronchoalveolar lavage was conducted by instilling 1 mL saline into the trachea, retaining the fluid for 60 s, and then withdrawing it. The collected bronchoalveolar lavage fluid (BALF) was immediately frozen at −80 °C.

### 2.5 Histopathological examination via H&E staining

Paraffin-embedded lung sections were H&E-stained for light microscopic analysis. Lung injury was scored blindly according to established guidelines (Kulkarni et al., 2022) by assessing key histopathological features across five random fields, with results normalized to a continuous 0–1 scale.

### 2.6 Lung wet/dry weight ratio

The wet/dry (W/D) weight ratio was employed to evaluate pulmonary edema. The inferior lobe of the right lung was dissected, weighed, oven-dried for 48 h at 60 °C and reweighed.

### 2.7 Cell culture

BEAS-2B cells were cultured in high-glucose DMEM supplemented with 10% FBS and 1% P/S under standard conditions. Cells were kept in logarithmic growth through regular passaging and medium changes every 2–3 days.

### 2.8 Cell treatments and grouping

Cells were assigned to six groups: ① NC; ② EGCG; ③ PM2.5; ④ PM2.5 + EGCG; ⑤ PM2.5 + T0070907 (PPAR-γ antagonist); ⑥ PM2.5 + EGCG + T0070907. Prior to PM2.5 challenge, groups ⑤ and ⑥ received 10 μM T0070907 for 2 h (Li Q. et al., 2023; Chen et al., 2023), while groups ②, ④, and ⑥ received a 12-h pretreatment with 50 μg/mL EGCG (Zhu et al., 2020). All groups, except NC, were subsequently treated with 100 μg/mL PM2.5 for 24 h (Liu et al., 2025).

### 2.9 Cytokine quantification

Concentrations of IL-1β, IL-6, and TNF-α in BALF, serum, and BEAS-2B supernatants were measured using ELISA according to manufacturer guidelines, with absorbance read at 450 nm.

### 2.10 Analysis of oxidative stress biomarkers

Tissue homogenates (10%) were prepared in saline (1:9 w/v) and centrifuged to obtain supernatants. Levels of malondialdehyde (MDA), lung tissue and BEAS-2B activities of CAT and SOD were measured using commercial assay kits. Intracellular ROS in treated BEAS-2B were assessed with the fluorescent probe DCFH-DA. Cells were loaded with DCFH-DA probe (diluted 1:1000) and incubated, washed, and imaged by fluorescence microscopy (488/525 nm). ROS levels were quantified based on fluorescence intensity using ImageJ.

### 2.11 Protein expression analysis by Western blotting

Protein expression of PPAR-γ, phospho-NF-κB (p-NF-κB), NF-κB and HO-1 was analyzed via Western blot. Protein extracts from lung and BEAS-2B were obtained using RIPA buffer with inhibitors and quantified by BCA. After electrophoresis and transfer, membranes were blocked, incubated sequentially with primary and HRP-secondary antibodies, washed with TBST, and developed using ECL.

### 2.12 Statistical analysis

Data are expressed as mean ± SD (n ≥ 3). Group differences were analyzed by one-way ANOVA with Tukey’s *post hoc* test, and *p* < 0.05 was considered significant.

## 3 Results

### 3.1 EGCG pretreatment ameliorates PM2.5-induced lung pathological damage

**FIGURE 1:**
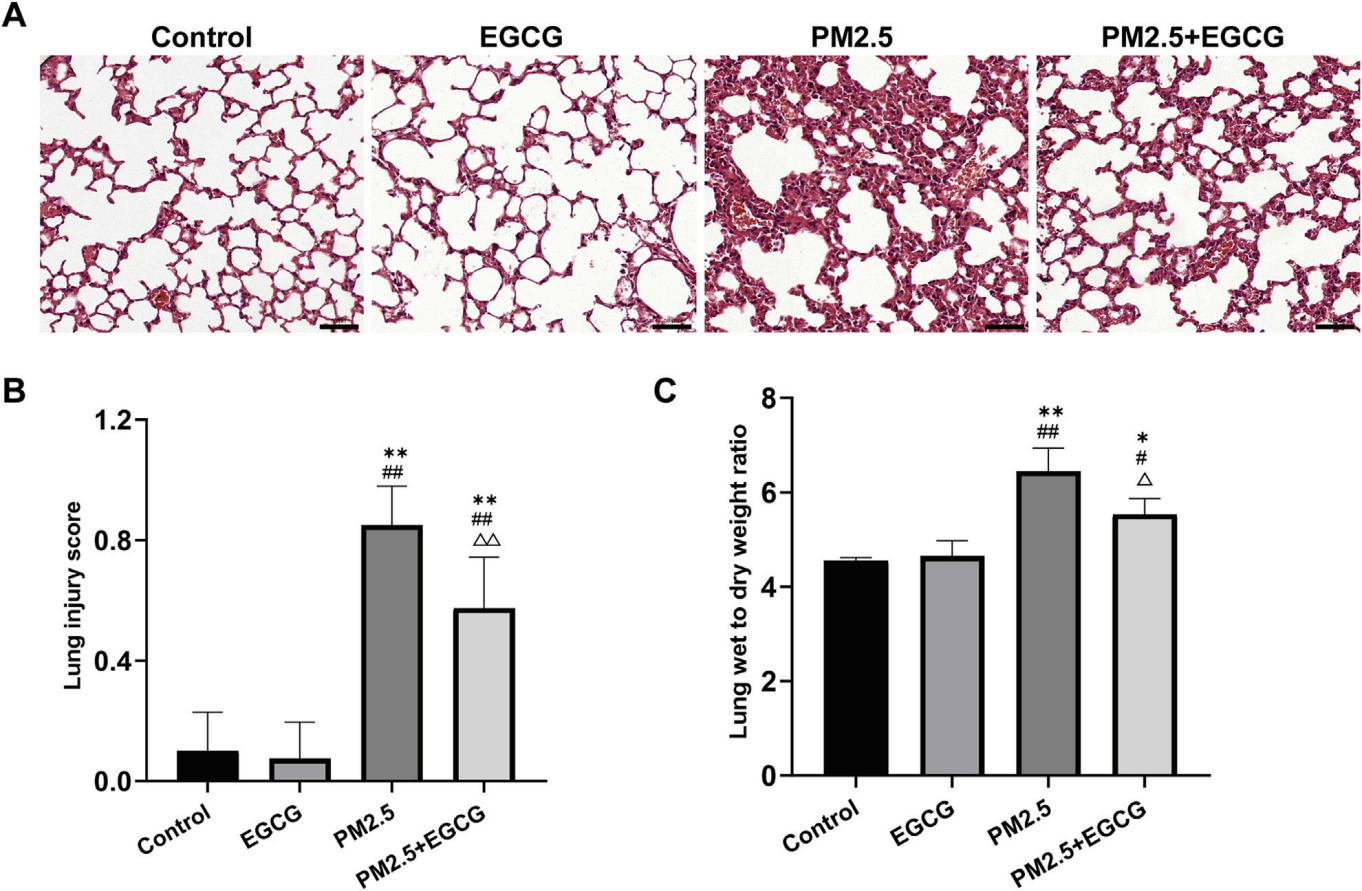
Pathological changes in lung tissues of mice. **(A)** HE staining. Scale bar = 50 μm. **(B)** Lung injury score. **(C)** Lung wet to dry weight ratio. Data are presented as mean ± SD. ^*^ *P* < 0.05, ^**^ *P* < 0.01 vs. Control group; ^#^ *P* < 0.05, ^##^ *P* < 0.01 vs. EGCG group; ^△^ *P* < 0.05, ^△△^ *P* < 0.01 vs. PM2.5 group.

Histopathological examination revealed that animals exposed to PM2.5 exhibited significant bronchial wall thickening, perivascular inflammatory cell infiltration, erythrocyte extravasation, and alveolar septa disruption. Quantitative evaluation revealed a significantly elevated lung injury score in PM2.5-exposed animals relative to both Control and EGCG groups, both of which maintained normal airway architecture without evident inflammation (Figures 1A,B). Pretreatment with EGCG significantly alleviated these pathological changes, demonstrating diminished inflammatory infiltration, thinner bronchial walls, diminished erythrocyte extravasation, and preserved alveolar structure (Figure 1A), culminating in a significant improvement in the histopathological injury score (Figure 1B).

### 3.2 EGCG attenuates PM2.5-induced pulmonary edema

The W/D ratios in the Control and EGCG groups remained within the normal range. Notably, the PM2.5 group showed a significant rise, whereas EGCG pretreatment notably lowered the W/D ratio relative to PM2.5-treated mice (Figure 1C), suggesting its efficacy in reducing pulmonary edema.

### 3.3 EGCG reduces PM2.5-inducedincreases in pro-inflammatory cytokines within BALF and serum

**FIGURE 2:**
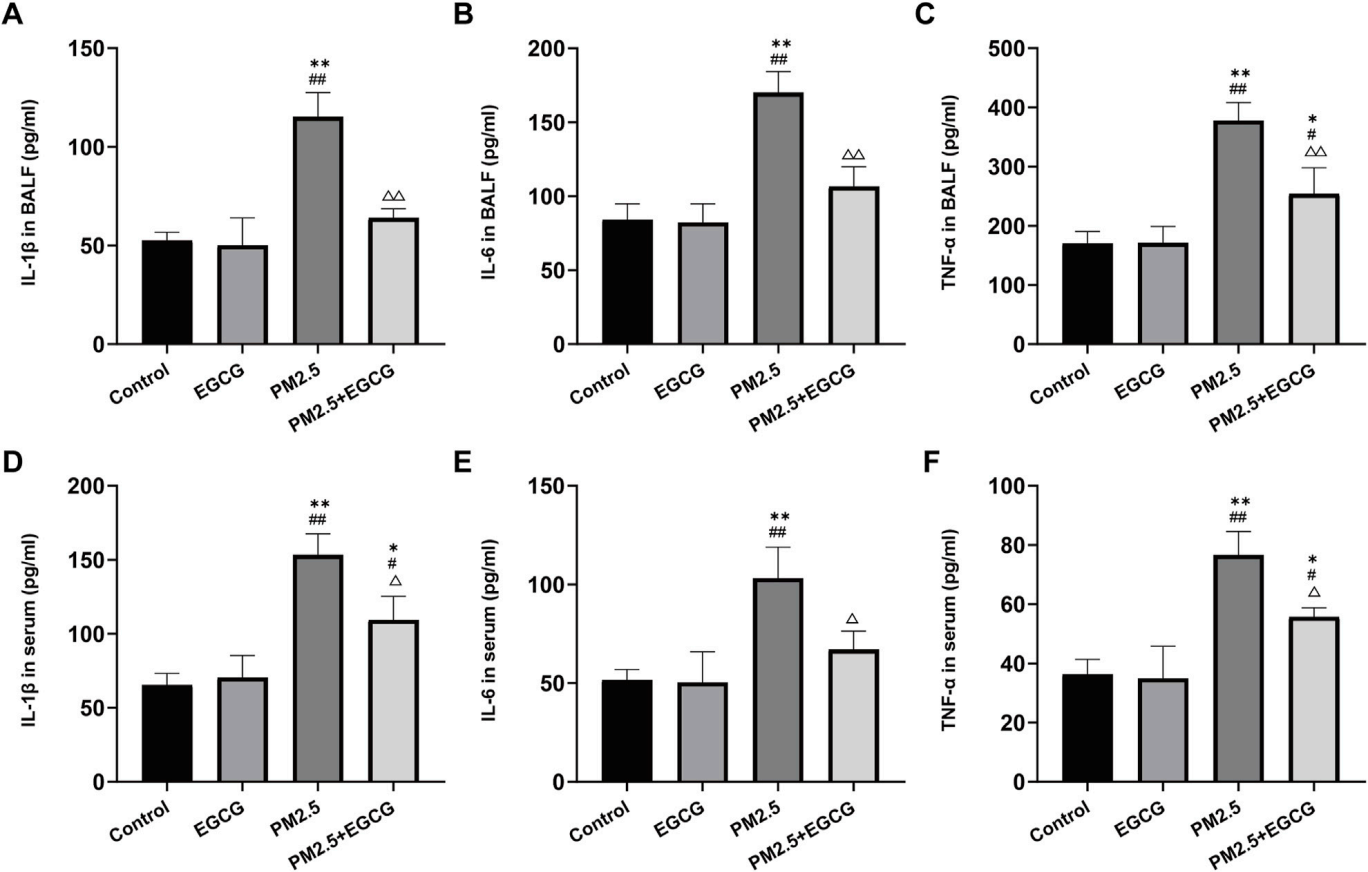
Effects of EGCG on inflammation in mice. **(A–C)** Levels of IL-1β, IL-6 and TNF-α in BALF. **(D–F)** Levels of IL-1β, IL-6 and TNF-α in serum. Data are presented as mean ± SD. ^*^ *P* < 0.05, ^**^ *P* < 0.01 vs. Control group; ^#^ *P* < 0.05, ^##^ *P* < 0.01 vs. EGCG group; ^△^ *P* < 0.05, ^△△^ *P* < 0.01 vs. PM2.5 group.

Concentrations of IL-1β, IL-6, and TNF-α remained low and comparable in both BALF and serum samples from the Control and EGCG-treated animals. PM2.5 led to a marked upregulation of these cytokine levels. In contrast, EGCG pretreatment substantially suppressed the PM2.5 triggered upregulation of these mediators (Figure 2).

### 3.4 EGCG attenuates PM2.5 triggered lung tissue oxidative imbalance

**FIGURE 3:**
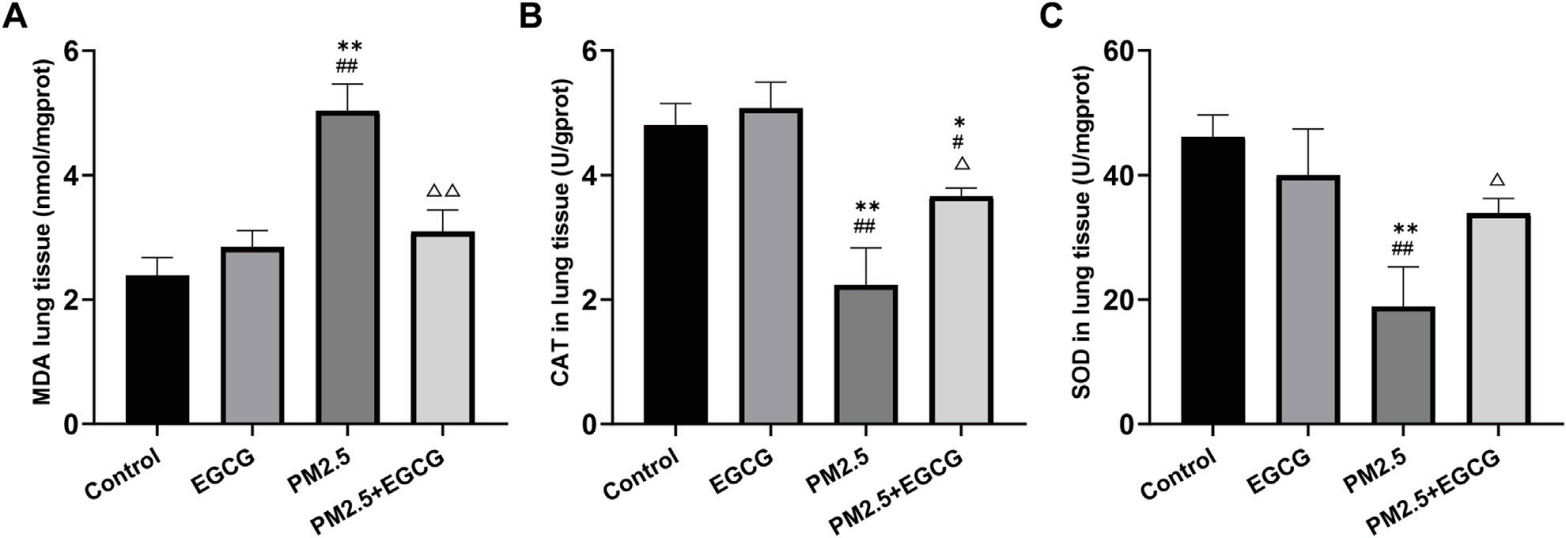
Effects of EGCG on oxidative stress in mice. **(A)** MDA levels in lung tissues. **(B)** SOD activities in lung tissues. **(C)** CAT activities in lung tissues. Data are presented as mean ± SD. ^*^ *P* < 0.05, ^**^ *P* < 0.01 vs. Control group; ^#^ *P* < 0.05, ^##^ *P* < 0.01 vs. EGCG group; ^△^ *P* < 0.05, ^△△^ *P* < 0.01 vs. PM2.5 group.

Low MDA levels alongside high CAT and SOD activities were observed in both Control and EGCG-treated animals, whereas PM2.5 exposure markedly elevated MDA and suppressed these antioxidant enzyme activities. In contrast, EGCG pretreatment effectively decreased MDA levels and restored the activities of these antioxidant enzymes (Figure 3), indicating that EGCG ameliorates PM2.5-induced oxidative imbalance.

### 3.5 EGCG pretreatment activates lung tissue PPAR-γ expression suppressed by PM2.5

**FIGURE 4:**
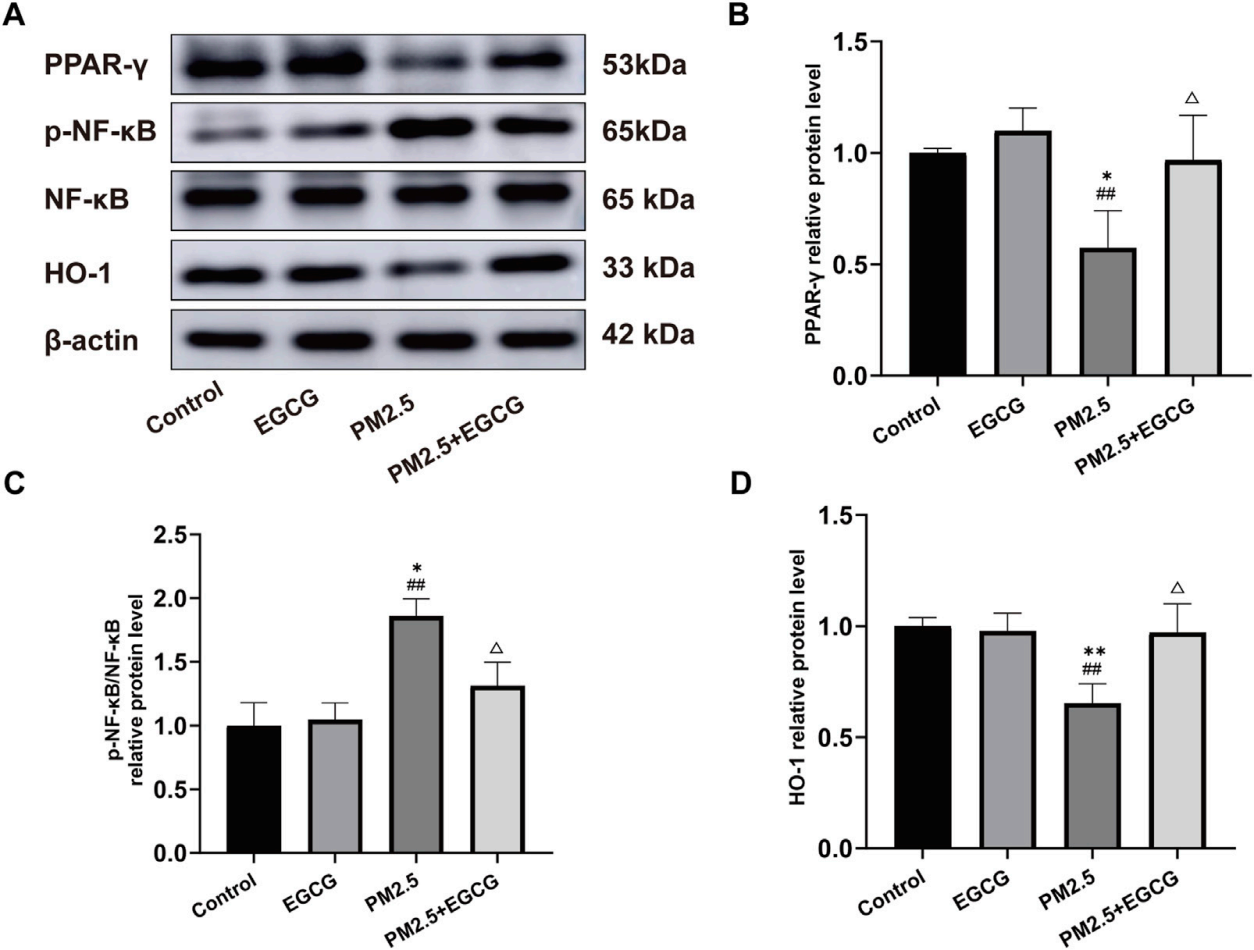
Effects of EGCG on the PPAR-γ pathway in lung tissues. **(A)** Protein expression of PPAR-γ, p-NF-κB, NF-κB and HO-1 in lung tissues. **(B–D)** Relative protein levels of PPAR-γ, p-NF-κB/NF-κB and HO-1 in lung tissues.·Data are presented as mean ± SD. ^*^ *P* < 0.05, ^**^ *P* < 0.01 vs. Control group; ^##^ *P* < 0.01 vs. EGCG group; ^△^ *P* < 0.05 vs. PM2.5 group.

Compared to the Control and EGCG groups, PM2.5 exposure significantly downregulated PPAR-γ expression, upregulated the p-NF-κB/NF-κB ratio and reduced HO-1 expression (Figure 4). These changes suggest NF-κB activation and HO-1 suppression under PM2.5-induced stress. EGCG pretreatment notably elevated PPAR-γ expression, decreased p-NF-κB/NF-κB ratio, and enhanced HO-1 levels (Figure 4), suggesting that EGCG may modulate PM2.5-induced alterations in the PPAR-γ pathway.

These findings suggest that EGCG attenuates PM2.5-triggered pulmonary damage in mice, potentially via modulating PPAR-γ signaling, which consequently alleviates inflammatory and oxidative responses.

### 3.6 EGCG attenuates PM2.5 triggered inflammation and oxidative damage in BEAS-2B

**FIGURE 5:**
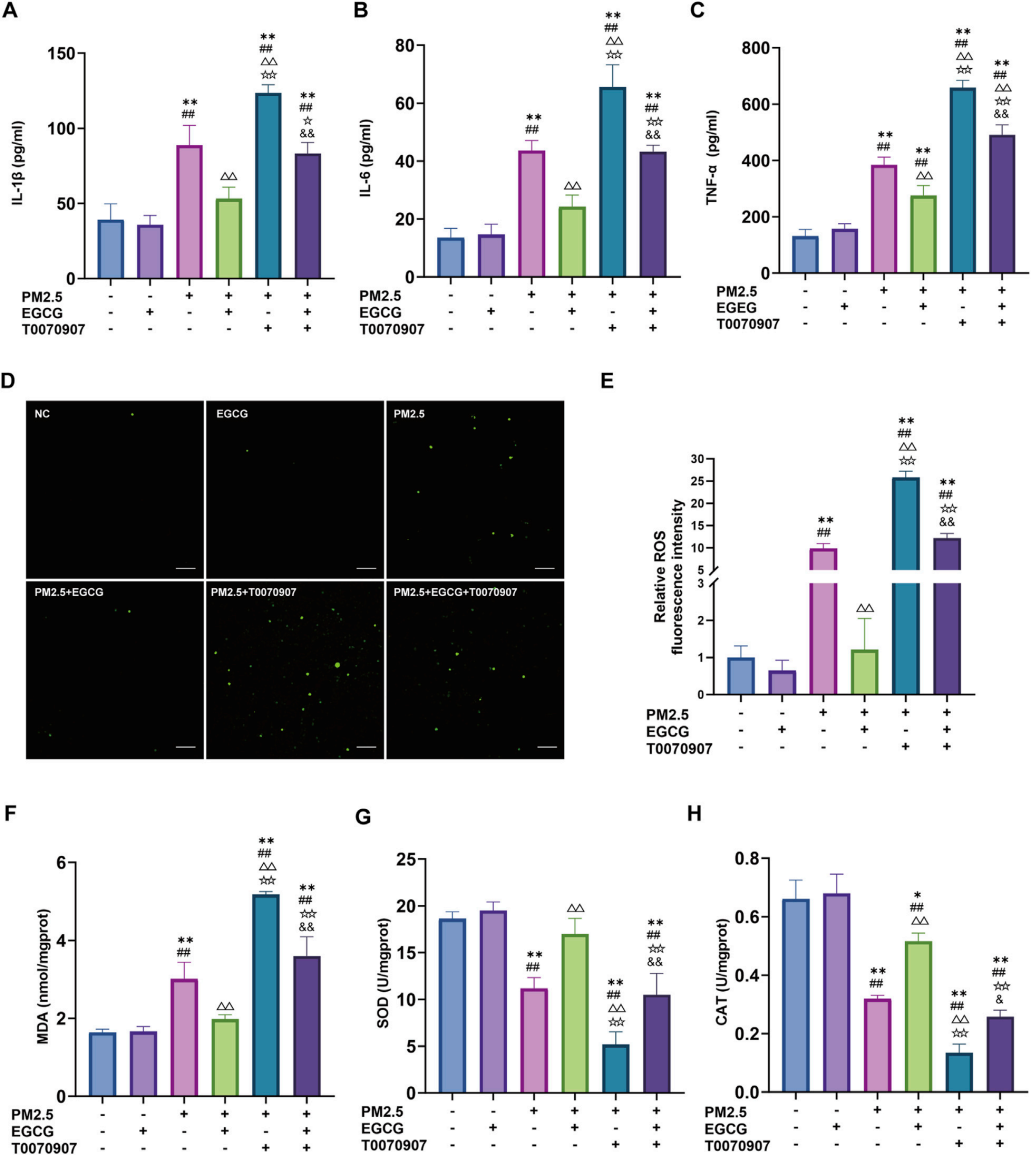
Effects of EGCG on inflammation and oxidative stress in BEAS-2B cells. **(A–C)** Levels of IL-1β, IL-6 and TNF-α in BEAS-2B cell supernatant. **(D,E)** Fluorescence intensity and quantification of ROS in BEAS-2B cells. Green represents DCFH-DA, Scale bar = 100 μm. **(F)** Intracellular MDA levels. **(G)** Intracellular SOD activities. **(H)** Intracellular CAT activities. Data are presented as mean ± SD. ^*^ *P* < 0.05, ^**^ *P* < 0.01 vs. NC group; ^##^ *P* < 0.01 vs. EGCG group; ^△△^ *P* < 0.01 vs. PM2.5 group; ^☆^ *P* < 0.05, ^☆☆^ *P* < 0.01 vs. PM2.5 + EGCG group; ^&^ *P* < 0.05, and ^&^ *P* < 0.01 vs. PM2.5 + T0070907 group.

To elucidate the EGCG’s protective mechanisms against PM2.5 related lung injury, *in vitro* studies were performed with BEAS-2B. Compared to the NC and EGCG groups, PM2.5 increased the IL-1β, IL-6, and TNF-α levels significantly (Figures 5A–C), elevated oxidative stress markers including ROS and MDA (Figures 5D–F), and suppressed CAT and SOD activities (Figures 5G,H). Treatment with EGCG effectively reversed these PM2.5-induced alterations, markedly reducing inflammatory cytokines and oxidative damage, while restoring antioxidant capacity (Figure 5).

### 3.7 EGCG modulates the PM2.5-suppressed PPAR-γ pathway in BEAS-2B

**FIGURE 6:**
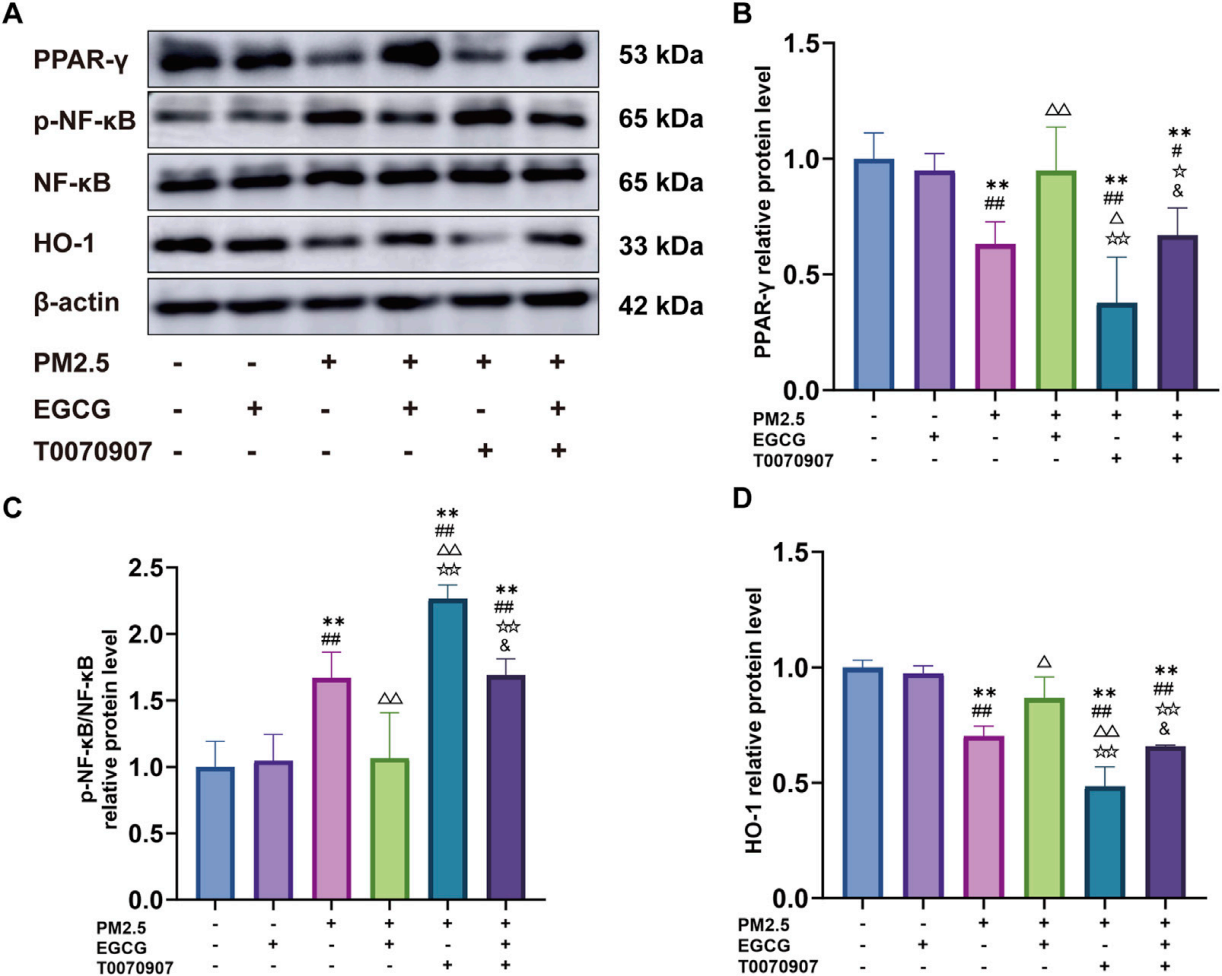
Effects of EGCG on the PPAR-γ pathway in BEAS-2B cells. **(A)** Protein expression of PPAR-γ, p-NF-κB, NF-κB and HO-1 in BEAS-2B cells. **(B–D)** Relative protein levels of PPAR-γ, p-NF-κB/NF-κB and HO-1 in BEAS-2B cells. Data are presented as mean ± SD. ^**^ *P* < 0.01 vs. NC group; ^#^ *P* < 0.05, ^##^ *P* < 0.01 vs. EGCG group; ^△^ *P* < 0.05, ^△△^ *P* < 0.01 vs. PM2.5 group; ^☆^ *P* < 0.05, ^☆☆^ *P* < 0.01 vs. PM2.5 + EGCG group; ^&^ *P* < 0.05 vs. PM2.5 + T0070907 group.

Relative to the NC and EGCG groups, PM2.5 exposure significantly downregulated PPAR-γ expression, increased the p-NF-κB/NF-κB ratio and decreased HO-1 expression, indicating activation of NF-κB and suppression of HO-1 (Figure 6). EGCG pretreatment effectively restored PPAR-γ expression, reduced the p-NF-κB/NF-κB ratio, and increased HO-1 levels (Figure 6), demonstrating that EGCG counteracts PM2.5-induced dysregulation of the PPAR-γ pathway and its downstream targets.

### 3.8 The protective role of EGCG is mediated through PPAR-γ signal

Administration of the PPAR-γ antagonist T0070907 markedly downregulated PPAR-γ levels in BEAS-2B, increased he p-NF-κB/NF-κB ratio, and inhibited HO-1 (Figure 6). These alterations exacerbated PM2.5-induced inflammatory activation, oxidative damage, and suppression of antioxidant enzyme activities (Figure 5). More importantly, the protective effects of EGCG were partially abolished by pharmacological inhibition of PPAR-γ with T0070907. Specifically, T0070907 attenuated the ability of EGCG to reduce IL-1β, IL-6, and TNF-α (Figures 5A–C), and diminished its ameliorative effects on oxidative stress markers (Figures 5D–F). Furthermore, EGCG’s restorative impact on catalase and SOD activities was significantly inhibited (Figures 5G,H).

## 4 Discussion

PM2.5 is a widespread airborne pollutant posing serious risks to respiratory health, yet treatment options for related lung injury remain limited. This study demonstrates that EGCG, a plant-derived polyphenol, mitigates pulmonary injury triggered by PM2.5 through attenuation of inflammatory and oxidative pathways. Rescue experiments with a PPAR-γ antagonist confirmed that this protection is partly dependent on PPAR-γ activation. These data underscore EGCG’s potential against air pollution-related lung injury.

Pulmonary damage triggered by PM2.5 is largely mediated by inflammatory pathways and disruption of redox homeostasis. Following exposure to PM2.5, mice develop pronounced inflammatory infiltration and structural alterations in lung epithelium, with tissue recovery requiring prolonged reparative mechanisms (Li Y. et al., 2023). Accumulating evidence indicates that in BEAS-2B and murine models, PM2.5 significantly upregulates pro-inflammatory cytokines and suppresses antioxidant capacity (Gao et al., 2022; Xie et al., 2022; Wang et al., 2025). Additionally, PM2.5 exacerbates mitochondrial dysfunction and apoptosis in lung tissues, processes also associated with inflammatory and oxidative pathways (Zhang et al., 2021). Our results align with these earlier findings, demonstrating that PM2.5 instigates lung injury characterized by inflammatory cell infiltration, disruption of alveolar architecture, and elevated levels of inflammatory mediators and oxidative markers. Specifically, intratracheal instillation of PM2.5 induced pulmonary edema, inflammatory infiltration, alveolar wall destruction, and dysregulation of cytokines and redox homeostasis in mice. Similarly, BEAS-2B with PM2.5 treatment also triggered pronounced secretion of pro-inflammatory cytokines and disrupted redox balance.

PPAR-γ, a ligand-activated nuclear receptor, regulates genes involved in metabolic balance, cellular differentiation, and inflammatory and oxidative pathways (Zhang et al., 2024). Traffic-related PM2.5 induced respiratory damage in mice and 16HBE cells via PPAR-γ-mediated systemic and local inflammatory responses, along with apoptotic activation (Gong et al., 2022). In acute lung injury models, PPAR-γ modulates inflammatory and barrier damage in pulmonary and colonic epithelium (Wang et al., 2024). Moreover, PM2.5 has been reported to drive inflammatory reactions and promote pulmonary fibrosis through downregulation of PPAR-γ and concurrent activation of the HMGB1/NLRP3 inflammasome pathway (Yang et al., 2024).

PPAR-γ and its downstream signaling molecules, particularly NF-κB and HO-1, play pivotal roles in modulating oxidative stress and inflammatory responses (Lee et al., 2024). In rat models of lung and intestinal injury, decreased PPAR-γ expression was accompanied by increased NF-κB activation (Gendy et al., 2021). Cigarette smoke exposure was shown to reduce PPAR-γ/HO-1 in murine lung tissue and alveolar macrophages, thereby attenuating inflammation and M1 polarization in a COPD model (Feng et al., 2024). In alignment with these reports, our study demonstrates that PM2.5 inhibits PPAR-γ protein expression, promotes NF-κB activation, and suppresses HO-1 signaling in both mouse lung tissues and BEAS-2B cells. These alterations collectively lead to increased release of inflammatory mediators and disruption of redox homeostasis, ultimately contributing to pulmonary injury. These findings highlight the essential involvement of PPAR-γ dysregulation in PM2.5 triggered lung pathology.

Plant-derived phytochemicals are increasingly investigated for mitigating PM2.5-induced toxicity due to their bioactivity and favorable safety. EGCG, a major green tea polyphenol, demonstrates strong antioxidative and anti-inflammatory activities (Guo et al., 2024). Accumulating evidence suggests a regulatory relationship between EGCG and PPAR-γ. EGCG has been shown to ameliorate various kidney diseases and improve renal pathology, mechanisms associated with PPAR-γ-mediated suppression of inflammation and oxidative stress (Kanlaya and Thongboonkerd, 2019). Additionally, EGCG can modulate key pathways such as PPAR-γ, influencing epigenetic mechanisms including DNA methylation and histone modifications, thereby attenuating age-related physiological decline through the regulation of free radicals, oxidative stress, and inflammatory responses (Al-Regaiey, 2024). EGCG also significantly inhibits adipocyte differentiation and lipid droplet accumulation, effects closely linked to PPAR-γ modulation (Oruganti et al., 2023). In microglial cells, EGCG upregulates HO-1 expression, reduces pyroptosis and neuroinflammation, promotes M1-to-M2 polarization, and alleviates neuroinflammation following intracerebral hemorrhage (Bao et al., 2023). Furthermore, EGCG pretreatment counteracts 6-OHDA-induced neurotoxicity in N27 cells by suppressing ROS generation, partly through mechanisms involving PPAR-γ and Nrf2/HO-1 (Xu Q. et al., 2024).

Within respiratory disease models, EGCG have shown protective effects against silica-induced lung damage through IL-17/NF-κB signaling (Xu Y. et al., 2024). EGCG reduces EGFR and suppresses HIF-1α, HK2, PKM2, and iNOS expression, thereby attenuating sepsis-associated acute lung injury (Jin et al., 2025). A recent study showed that EGCG pretreatment significantly suppressed inflammatory cell counts, cytokine expression, ROS generation, and neutrophil extracellular trap formation in mouse lungs exposed to urban aerosols, though the precise mechanisms require further investigation (Tanaka et al., 2022). Whereas previous reports primarily describe EGCG’s protective outcomes, our work provides a novel mechanistic depth by delineating the essential role of the PPAR-γ pathway in mediating these effects specifically against PM2.5 insult. Our findings are consistent with these reports and extend them mechanistically. We demonstrated that EGCG significantly alleviated pulmonary edema and histopathological damage triggered by PM2.5 in mice, and reduced inflammatory cytokines and oxidative damage. Mechanistically, EGCG pretreatment upregulated PPAR-γ expression, subsequently inhibiting NF-κB activation and enhancing HO-1 activity. Importantly, pharmacological inhibition of PPAR-γ with T0070907 partially reversed the protective effects of EGCG, as evidenced by the renewed inflammatory response and elevated oxidative stress. These functional data demonstrate that PPAR-γ activation plays an essential role in the protective mechanism of EGCG against PM2.5-mediated lung injury.

This study has several limitations that should be considered. First, while pharmacological inhibition confirms the functional importance of PPAR-γ, our data cannot distinguish whether EGCG directly activates the receptor or indirectly modulates it through improving cellular homeostasis. Second, the partial reversal of EGCG’s protection by the PPAR-γ antagonist suggests that additional, PPAR-γ-independent pathways may contribute to its overall efficacy. Future studies employing direct binding assays, reporter gene systems, and antagonist dose-response curves will be valuable to precisely elucidate the mechanism of PPAR-γ activation and fully delineate its contribution to EGCG’s protective effects.

## 5 Conclusion

In summary, our findings suggest that EGCG may protect against PM2.5-induced lung injury by modulating the PPAR-γ pathway, leading to reduced NF-κB activation and enhanced HO-1 expression. This study supports the potential of EGCG as a candidate for further investigation into preventive strategies for air pollution-related respiratory damage.

## Data Availability

The original contributions presented in the study are included in the article/Supplementary Material, further inquiries can be directed to the corresponding authors.
