# Supplementary material for: EGCG alleviates PM2.5-induced lung injury via activation of PPAR-γ to suppress inflammation and oxidative stress
Source: Front Pharmacol. 2025 Nov 13;16:1695785. doi: 10.3389/fphar.2025.1695785 (PMC12657361; doi:10.3389/fphar.2025.1695785)
Supplement: Supplementary file 1 [file Table1.docx]

Supplementary Material 1

**Table S1** Commercial kits used in this study

| **Antibody** | **Manufacturer** |
| --- | --- |
| IL-1β ELISA kits | Enzyme Immunoassay Biotech |
| IL-6 ELISA kits | Enzyme Immunoassay Biotech |
| TNF-α ELISA kits | Enzyme Immunoassay Biotech |
| malondialdehyde (MDA) | Nanjing Jiancheng Bioengineering Institute |
| catalase (CAT) | Nanjing Jiancheng Bioengineering Institute |
| total superoxide dismutase (SOD) | Nanjing Jiancheng Bioengineering Institute |
| reactive oxygen species (ROS) | Servicebio Technology |

**Table S2** Antibodies used in western blot

| **Antibody** | **Manufacturer** |
| --- | --- |
| PPAR gamma (cat: P011295) | Epizyme Biotech |
| Phospho-NF-κB p65 (cat: #3033) | Cell Signaling Technology |
| NF-κB p65 (cat: 80979-1-RR) | Proteintech Group |
| Heme Oxygenase 1 (cat: R014764) | Epizyme Biotech |
| β-actin (cat: 20536-1-AP) | Proteintech Group |
| HRP-conjugated goat anti-rabbit IgG (cat: SA00001-2) | Proteintech Group |

**Table S3** Composition of inorganic metallic elements in PM2.5 samples (n = 6)

| **Inorganic metallic elements** | **Concentration (μg/m³)** |
| --- | --- |
| Na | 0.05 (0.00 - 0.53) |
| Mg | 0.21 (0.11 - 0.40) |
| Al | 0.24 (0.10 - 0.46) |
| Si | 0.42 (0.10 - 0.84) |
| S | 0.74 (0.04 - 1.86) |
| Cl | 0.42 (0.10 - 1.36) |
| K | 0.34 (0.27 - 0.61) |
| Ca | 0.39 (0.15 - 0.78) |
| Ti | 0.16 (0.00 - 0.61) |
| Cr | 0.00 (0.00 - 0.01) |
| Mn | 0.02 (0.01 - 0.06) |
| Fe | 0.74 (0.14 - 0.89) |
| Cu | 0.05 (0.00 - 0.26) |
| Zn | 0.03 (0.02 - 0.17) |
| As | 0.00 (0.00 - 0.02) |
| Br | 0.00 (0.00 - 0.02) |
| Sr | 0.01 (0.00 - 0.01) |
| Pb | 0.01 (0.00 - 0.06) |

Data are presented as medians (min-max).

**Table S4** Water-soluble inorganic ions in PM2.5 samples (n = 6)

| **Water-soluble inorganic ions** | **Concentration (μg/m³)** |
| --- | --- |
| F^-^ | 0.22 (0.03 - 0.48) |
| Cl^-^ | 1.44 (0.04 - 5.35) |
| NO_3_^-^ | 8.05 (1.19 - 17.05) |
| SO_4_^2-^ | 8.13 (3.19 - 13.00) |
| Na^+^ | 0.31 (0.11 - 0.70) |
| NH_4_^+^ | 5.07 (1.16 - 10.35) |
| K^+^ | 0.39 (0.14 - 8.61) |
| Mg^2+^ | 0.10 (0.04 - 1.11) |
| Ca^2+^ | 1.35 (0.37 - 1.70) |

Data are presented as medians (min-max).

**Table S5** Composition of carbon components in PM2.5 samples (n = 6)

| **Composition of carbon** | **Concentration (μg/m³)** |
| --- | --- |
| Total Carbon (TC) | 8.03 (3.05 - 19.39) |
| Organic Carbon (OC) | 6.44 (2.55 - 15.68) |
| Elemental Carbon (EC) | 1.62 (0.50 - 3.71) |

Data are presented as medians (min-max).

**Table S6** Carbon component ratio in PM2.5 samples (n = 6)

| **Ratio** | **Value** |
| --- | --- |
| OC/EC | 3.98 (2.98 - 5.10) |

Data are presented as medians (min-max).
